# Cannabidiol and epilepsy in Brazil: a current review

**DOI:** 10.1590/0004-282X-ANP-2022-S137

**Published:** 2022-08-12

**Authors:** Carlos André Oshiro, Luiz Henrique Martins Castro

**Affiliations:** 1 Universidade de São Paulo, Faculdade de Medicina, Hospital das Clínicas, Departamento de Neurologia, São Paulo, SP, Brazil.

**Keywords:** Cannabidiol, Epilepsy, Efficacy, Safety, Brazil, Canabidiol, Epilepsia, Eficácia, Segurança, Brasil

## Abstract

**Background:**

Cannabidiol (CBD) has become a promising therapeutic option in the treatment of epilepsy. Recent studies provide robust evidence that CBD is effective and safe. Limitations in current knowledge and regulatory issues still limit CBD use. CBD use regarding epilepsy types still lacks clear guidelines.

**Objective:**

To critically review the main current pharmacological features and clinical issues regarding CBD use in epilepsy, to provide current regulatory background regarding CBD use in Brazil, and to suggest a practical CBD therapeutic guide in Brazil.

**Methods:**

Non-systematic literature review (up to February 2022) of current concepts of CBD and epilepsy, including the authors’ personal experience.

**Results:**

Five pivotal trials have led to CBD approval as an adjunctive treatment for Dravet and Lennox-Gastaut syndromes, and for the tuberous sclerosis complex. Efficacy of CBD in other drug-resistant epilepsies remains not completely understood. CBD adverse event profile and drug interactions are better understood. CBD is well tolerated. In Brazil, CBD is not classified as a medication, but as a product subject to a distinct regulatory legislation. CBD is still not offered by the National Brazilian health system, but can be purchased in authorized pharmacies or imported under prescription and signed informed consent.

**Conclusion:**

CBD is a recognized novel treatment for epilepsy. Future well-designed studies and public health strategies are needed to offer widespread access to CBD, and to improve the quality of life of people living with epilepsy in Brazil.

## RESUMO

**Antecedentes:**

O canabidiol (CBD) é uma terapêutica promissora no tratamento da epilepsia. Estudos recentes trouxeram evidências robustas sobre a eficácia e segurança do CBD. Lacunas no conhecimento atual e questões legais limitam seu uso. Não há consenso sobre o manejo do CBD nos diferentes tipos de epilepsia.

**Objetivo:**

Revisar criticamente os principais aspectos farmacológicos e clínicos atuais do uso do CBD em epilepsia e sugerir um guia terapêutico prático no Brasil.

**Métodos:**

Revisão não-sistemática da literatura até fevereiro de 2022 de conceitos atuais sobre CBD e epilepsia, associado a experiência dos autores.

**Resultados:**

Cinco estudos principais levaram à aprovação do CBD como tratamento adjuvante nas síndromes de Dravet, Lennox-Gastaut e na esclerose tuberosa. A eficácia em outras epilepsias fármaco-resistentes ainda não está bem estudada. Eventos adversos e interações medicamentosas são comuns. O CBD é bem tolerado. No Brasil, o CBD não é classificado como medicamento, mas como produto sujeito a diferente regras regulatórias. O acesso ao CBD ainda é restrito, e este não é fornecido pelo sistema público de saúde brasileiro. O CBD pode ser adquirido no Brasil em farmácias autorizadas ou por importação, sob prescrição especial e termo de consentimento informado.

**Conclusão:**

O CBD é uma realidade no tratamento da epilepsia. Estudos futuros e políticas públicas são necessários para permitir amplo acesso do CBD melhoraria da qualidade de vida de pessoas com epilepsia no Brasil.

## INTRODUCTION

Approximately 50 million people live with epilepsy worldwide^1^. Epilepsy is not a single disease, but an array of neurological disorders sharing an enduring predisposition to generate epileptic seizures^2^. Social, physical, psychological, and economic disease substantially create an impact on affected individuals, their families and caregivers, as well as on society as a whole^3^.

The development of drugs with novel mechanisms to suppress seizure occurrence has doubled in the past decades, but the proportion of patients with medically refractory disease has not decreased significantly^4^. Up to 30% of patients with epilepsy still present persistent uncontrolled seizures, despite adequate trials of two efficacious and tolerated antiseizure medications (ASM), and this characterizes drug-resistant epilepsy^5^ ^,^ ^6^. 

In 2013, medical use of *Cannabis sativa* attracted considerable public attention after the successful broadcast experience of Charlotte Figi in the United States. The five-year girl with Dravet syndrome had a more than 90% seizure reduction after using a high-CDB-strain cannabis extract^7^. The therapeutic use of the Cannabis plant (also known as marijuana) has been reported for centuries in anecdotal reports^8^. Since the mid 20^th^ century, more intensive research on cannabidiol (CBD) and delta-9-tetrahydrocannabinol (THC) *Cannabis sativa* compounds have provided evidence of their efficacy in treating epilepsy and other neurological conditions^9^. A better characterization of the endocannabinoid system also expanded the therapeutic horizon of these compounds. CBD was shown to have a powerful anti seizure effect without psychoactive effects, compared to THC^9^ ^,^ ^10^. 

**Figure 1.**
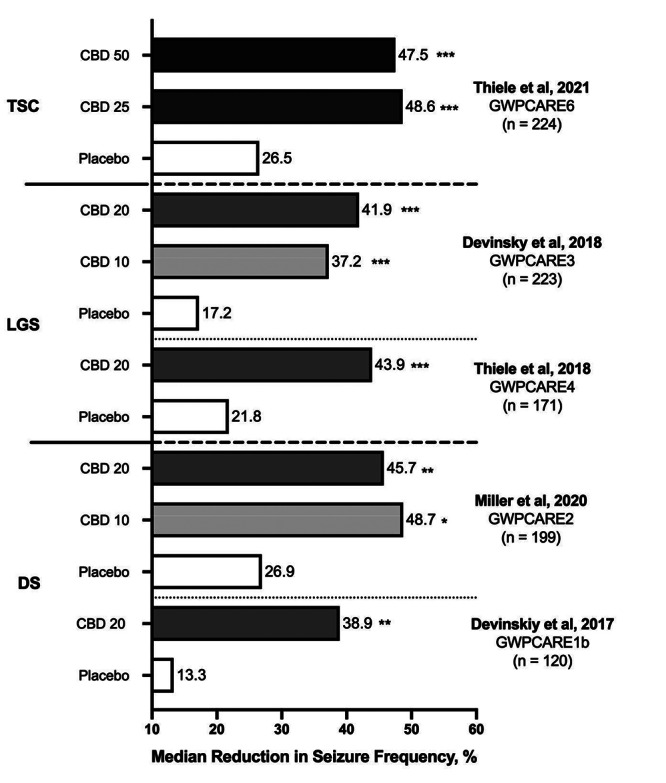
CBD efficacy. Reduction in seizure frequency during the 14-week-treatment of the five randomized clinical trials with cannabidiol in patients with Tuberous Sclerosis Complex (TSC), Lennox-Gastaut Syndrome (LGS) and Dravet Syndrome (DS). Doses: 10 mg/kg/d (CBD 10), 20 mg/kg/d (CBD 20), 25 mg/kg/d (CBD 25) and 50 mg/kg/d (CBD 50).

^*^P < 0.05; ^**^P ² 0.01; ^***^ P ² 0.005 displays the comparisons between CBD groups and corresponding placebo groups.

In 2014, a systematic review from Cochrane and the American Academy of Neurology showed lack of evidence to support the use of cannabidiol (CBD) for epilepsy^11^. The four clinical trials then available presented methodological limitations that underpowered analysis^12^ ^-^ ^15^. Subsequently, five randomized clinical trials, published between 2017 and 2021, showed evidence of efficacy and safety of CBD in epilepsy related to Dravet syndrome (DS), Lennox Gastaut syndrome (LGS), and in the tuberous sclerosis complex (TSC)^16^ ^-^ ^20^ (Figure 1), leading to approval of the highly purified CBD, *Epidiolex (GW Pharmaceuticals)* by the U.S. Food and Drug Administration (FDA)^21^ and by the European Medicines Agency (EMA)^22^ as an add-on treatment for these conditions, setting an important milestone in CBD use in epilepsy treatment. However, there are still no randomized clinical trials that support CBD use in other treatment-resistant epilepsy etiologies. Currently, only low-level open-label studies, using heterogeneous compositions, suggest a possible efficacy in these epilepsy types^23^. 

## CANNABIDIOL CLINICAL PHARMACOLOGY

CBD molecular structure was described by Mechoulan in 1963^24^. The endocannabinoid complex system was characterized in the 1990s, when cannabinoid types 1 (CB1) and 2 (CB2), as well as endogenous ligands anandamide and 2-arachidonoylglycerol - so-called endocannabinoids - were identified^8^. CBD anti-seizure action mechanism remains not fully understood. CBD displays no effect on cannabinoid receptors (CB1 and CB2), compared to THC, and therefore has no psychotropic effects^8^. Putative main mechanisms of CBD antiseizure action include modulation of intracellular calcium (via G protein-coupled GPRR55 receptors, transient receptor potential vanilloid type 1 TRPV1 channels, and voltage-dependent anion-selective channel protein 1 - VDAC1), leading to decreased neuronal excitability. Additionally, CBD influences anti-inflammatory adenosine-associated signaling pathways via equilibrative nucleoside transporter 1 - ENT-1 - inhibition and tumor necrosis alpha factor - TNF-alpha - so resulting in an antiepileptogenic effect^8^ ^,^ ^25^ ^,^ ^26^. 

 Preclinical cannabinoid studies in acute and chronic epilepsy animal models initiated in the 1970s showed a potential antiepileptic effect^27^. At that time, the proportion of cannabinoid and non-cannabinoid components in the extract was unknown. When CBD was later isolated, evidence became available of CBD antiepileptic properties in *in vitro* and *in vivo* experimental models^28^ ^,^ ^29^. 

 Initial studies in humans have led to a better understanding of CBD pharmacokinetics and pharmacodynamics^30^. The most common administration route - recreational inhalatory with pulmonary absorption - was transitioned to the oral route, using an oil-based capsule, which would be more suitable for studies and for future market distribution as medication. However, oral biodisponibility of the oral compound was poor, due to low gastrointestinal absorption related to CBD lipophilic properties, and to first-pass liver effect. All the same, a rapid absorption peak serum level occurs after two hours after ingestion^31^. Lipophilic properties and high protein affinity allow rapid and high CBD distribution in the nervous system and in other tissues. CBD accumulation in adipous tissues in chronic use remains a concern. CBD should preferably be taken with meals. Intranasal and transdermal routes are still under investigation^32^ ^,^ ^33^. 

CDB metabolism occurs almost exclusively in the liver, through cytochrome P450 enzymes. CPY2C219 hydrolyzes CBD to 7-hydroxy-cananbidiol (7-OH-CBD), followed by CYP3A4 9-hydroxy-cannabidiol (7-OH-CBD) formation. 7-OH-CBD is an inactive metabolite, eliminated in the stools, and to a lesser extent in urine. CBD has a half-life of 18 to 32 hours, allowing a twice-a-day dose regimen^34^. One third of patients present tolerance, requiring dose increases. CBD strongly inhibits CYP2C19 and CYP3A4. Drug interactions are a relevant matter in polytherapy scenarios^35^ (Table 1). 

**Table 1.** Most used anti-seizure medications (ASMs) pharmacokinetic interaction with cannabidiol (CBD). No interaction was observed of CBD with carbamazepine, clonazepam, ethosuximide, lamotrigine, midazolam, phenytoin, vigabatrin.

ASMs	Type of Evidence	Effect of CBD on ASMs	Effect of ASM on CBD
Clobazam	RCT	↑ [N-CLB]	↑ [7-OH-CBD]
Gabapentin	Preclinical	↑	↔
Lacosamide	Preclinical, observational	↑ (preclinical) ↔ (observational)	↑ ((preclinical)
Levetiracetam	Preclinical, RCT	↔ (preclinical, RCT)	↔ (preclinical)
Oxcarbazepine	Preclinical, RCT, observational	↑ (preclinical) ↔ (observational)	↑ (preclinical)
Phenobarbital	Observational	↔ / ↑	no studies
Stiripentol	RCT	↔ / ↑	?#8595;
Topiramate	Preclinical, RCT, observational	↑ (observational, preclinical) ↔ (RCT)	↑ (preclinical)
Valproate	Preclinical, RCT, observational	↔	↔

↑ elevation of plasma concentration; ↔ no change in plasma concentration; ?#8595; decrease in plasma concentration; CBD: cannabidiol; RCT: Randomized controlled trial; ASM: Anti-seizure medication.

CBD and clobazam (CLB) interactions are well recognized and relevant in clinical practice. Clobazam is metabolized by the P450 cytochrome complex: CYP3A4 metabolizes CLB to the active N-methyl clobazam (N-CLB) metabolite, and CYP2C19 metabolizes N-CLB to an inactive metabolite. CBD inhibits CYP2C19, resulting in increased N-CLB. Likewise, CLB increases CBD 7-hydroxy-cannabidiol, without increasing CBD levels. These interactions lead to increased side effects, such as sedation, drowsiness and fatigue. A meta analysis study demonstrated that CBD anti seizure effect occurred whether or not associated with CLB^36^. CBD action may be synergistic in the GABA-A receptor, with a possible action on GPR55 receptor^37^.

 CBD- valproate interaction is also clinically relevant. CBD does not influence VPA levels, and VPA does not influence CBD pharmacokinetics. Clinical studies showed increased liver enzymes, especially when CBD and VPA were used in association, possibly through potentialization of hepatotoxic effect in the mitochondria^38^. 

 Stiripentol, an antiseizure medication used for Dravet Syndrome (in addition to CLB and VPA) is also a CYP inhibitor, leading to CLB and N-CLB increases. Stiripentol does not significantly influence CBD pharmacokinetics, leading to decreased CBD metabolites. Stiripentol levels are mildly increased, when used in combination with CLB^38^.

Drug-to-drug interactions must also be considered not simply for anti seizure medications. Rapamycin inhibitors, used to treat subependymal giant cell astrocytomas and renal angiomyolipomas in tuberous sclerosis, in association with CBD, may lead to increased sirolimus, tacrolimus and everolimus levels, through CYP3A4 inhibition^39^. Methadone^40^ and warfarin^41^ levels are increased when used in combination with CBD. 

## EFFICACY AND SAFETY IN CLINICAL TRIALS

### Dravet syndrome (DS)

Dravet syndrome is a childhood onset epileptic encephalopathy. Most patients have a loss-of-function mutation in the voltage gated sodium channel α1 gene (SCN1A gene), and present prolonged focal and generalized seizures (absences, tonic-atonic, myoclonic and tonic-clonic seizures). Developmental delay and psychomotor changes are commonly seen. Two large clinical trials of CBD use in DS were completed - GWCAR1b^16^, in 2017 and GWCARE2, in 2020^19^. 

GWCAR1b enrolled 120 children and adolescents to evaluate efficacy as an add-on medication against placebo of a 20 mg/kg/d CBD dose at 14 weeks. Mean convulsive seizure reduction at 14 weeks of treatment was 38.9% versus 13.3% in the control group (p=0.01). There was a high rate of side effects in the CBD group (93%), compared to 75% in the placebo group. Eighty nine percent of side effects were deemed mild to moderate. Most common side effects were drowsiness, diarrhea, hyporexia and fatigue. Drug discontinuation due to side effects was 13% in the CBD group versus 2% in the placebo group^16^.

GWCARE2 studied 199 children using a similar protocol in three groups: CBD 10 mg/kg/d, CBD 20 mg/kg/d and placebo, in an add-on regimen. Mean seizure reduction was 48.7%, 45.7% and 26.9% respectively, statistically significant for 20 mg/kg (p= 0.03), and for 10mg/kg (p= 0.01), compared to placebo. An elevated rate of side effects was reported for all groups: 89.9% in the 20mg/kg group, 87.5% in the 10mg/kg group, and 89.2% in the placebo group. Ninety two percent of side effects were mild to moderate. Withdrawal rate was higher in the CBD 20mg/kg/d (9%), compared to 4.5% in the CBD 10 mg/kg/day, and zero in the placebo group. The most common side effects were hyporexia, diarrhea, drowsiness and fatigue. The highest incidence of drowsiness and pneumonia was noted with concomitant clobazam use. Elevated liver enzymes were seen in 12% of patients, all of them using valproate^19^.

An interim analysis of the long-term three year study, GWPCARE5, was published in 2021. This was an open-label trial that enrolled 315 of the patients that had participated in GWPCARE1b and GWPCARE2. GWPCARE5 analyzed efficacy data at 156 weeks, and safety data at 203 weeks. The mean CBD dose was 22 (range 20-24) mg/kg/day, and efficacy in reducing drop seizures was sustained at 156 weeks: 83% of patients reported an improved global condition during the whole follow-up period. Approximately 97% of patients reported side effects. In 72% of those, side effects were mild to moderate. Most relevant severe side effects were status epilepticus, convulsion, pneumonia and fever. Increased liver enzymes (three times or more above the upper limit of normal) was seen in 22% of patients (84% of whom with concomitant valproate use). Abnormal values were reverted in 86% of cases. Withdrawal rate was 45%. The main reasons for withdrawal were patient/guardian decision (19%) and side effects (8%)^42^.

*Post hoc* analysis of GWPCARE1b and GWPCARE2 319 patients was published in 2021 and evaluated time-to-efficacy and side effect duration. CBD led to significant seizure frequency reduction at two weeks of treatment (bearing in mind that all patients attained a 10 mg/kg/day dose at day seven in both studies). Fifty two percent of patients displayed side effects within two weeks of dose titration. Most common side effects were drowsiness, hyporexia and diarrhea, which were resolved within four weeks in most patients^43^. 

### Lennox-Gastaut syndrome (LGS)

Lennox-Gastau is a childhood onset epileptic encephalopathy characterized by significant cognitive impairment, multiple seizure types, and abnormal EEG findings - background slowing and slow-spike and wave complexes. Two randomized studies of CBD effect in an add-on regimen on drop seizures in LGS - GWPCARE3 (17) and GWPCARE4^18^ were published in 2018.

GWPCARE3 enrolled 223 patients, randomized to 10 mg/kg/d, 20 mg/kg/d and placebo. Primary endpoint with median drop seizure reduction was 37.2, 41.9 e 17.2% after 14 weeks of treatment respectively, statistically significant for 20 mg/kg (p= 0.005) and for 10mg/kg (p=0.002), compared to placebo. A high incidence of side effects was noted in all groups: 94% in the 20mg/kg group, 84% in the 10mg/kg group and 74% in the placebo group. Eighty nine percent of side effects were mild to moderate. Most common side effects were drowsiness, hyporexia and diarrhea. Treatment drop-out rates were low: 1.5% in the 10mg/kg/d, 7.3% in the 20 mg/kg/d, and 1.3% in the placebo group. A 50% discontinuation rate was attributed to medication side effects, especially for important transaminase elevation^17^. 

GWPCARE4 enrolled 171 patients randomized to CDB 20 mg/kg/d and placebo. Primary endpoint with median drop seizures reduction was 43.9% and 21.8% (p= 0.0135) at 14 treatment weeks, respectively. Side effects occurred in 86% of the intervention group and 69% of the control group. Eighty six percent of side effects were mild to moderate. Most common side effects were diarrhea, drowsiness, pyrexia, hyporexia, and vomiting. Treatment withdrawal was higher than in the other randomized controlled trial: 16% in the intervention group, and 1.2% in the placebo group. Eighty five percent of discontinuation was attributed to side effects, especially to significant increase in transaminase levels^18^.

 The final three-year long-term analysis - GWPCARE5 - an open-label trial enrolling 366 of LGS patients that participated in GWPCARE3 and GWPCARE4 was published in 2021. Efficacy data was evaluated at 156 weeks and safety data at 203 weeks. Median dose was 24 (range 2.5-30) mg/kg/d, and effectiveness in drop seizure reduction was sustained at 156 weeks. Eighty seven percent of patients reported improved overall conditions during the whole follow-up period. Ninety six percent of patients reported side effects, of which 68% were classified as mild and moderate. Most important severe side effects were convulsion, status epilepticus and pneumonia. Increased transaminases three times above the upper limit of normal was noted in 15% of patients, of whom 73% were taking concomitant valproate. Values reverted to normal in 95% of cases. Withdrawal rate was 33%. Main causes were attributed to patient/guardian decision (13%) and side effects (10%)^44^.

A post-hoc analysis of time to efficacy and side effect duration in the 396 patients participating in GWPCARE3 and GWPCARE4 was published in 2021. CBD led to significant reduction of drop seizure frequency at six days after initiating treatment (bearing in mind that all patients attained at least 10 mg/kg/d dose at day seven in both studies). Forty five percent of patients reported side effects within two weeks of dose titration. Most common side effects were drowsiness, hyporexia and diarrhea, which was resolved within four weeks in more than half of the patients^45^.

### Tuberous sclerosis complex (TSC)

Tuberous sclerosis is an autosomal dominant disease due to a TSC1 and/or TSC2 gene mutation leading to rapamycin (mTOR) pathway upregulation, predisposing to benign tumors (eye, skin, heart, kidney, lung and brain). A randomized controlled trial of CBD in an add-on regimen in epilepsy related to the TSC complex - GWPCARE6 - was published in 2020^20^.

GWPCARE6 enrolled 224 patients randomized to CDB 25 mg/kg/d, 50 mg/kg/d and placebo. The primary outcome, mean seizure reduction at 14 weeks of treatment, was 48.6, 47.5 and 26.5%, respectively, statistically significant for 25 mg/kg (p< 0.001) and for 50 mg/kg (p=0.002), compared to placebo. A high incidence of side effects was reported for all groups: 93% for the 25 mg/kg group, 100% for the 50mg/kg group and 95% for the placebo group. Eighty eight percent of side effects were considered mild to moderate. Most common side effects were diarrhea, drowsiness and hyporexia. Treatment withdrawal was low: 10.7% in the no 25 mg/kg/d group, 13.7% in the 50 mg/kg/d group and 2.6% in the placebo group. Treatment discontinuation was attributed to medication side effects in 78% of cases, importantly related to transaminase elevation^20^.

 A post-hoc analysis GWPCARE6 evaluated 224 patients regarding time to efficacy and side effect duration. CBD led to important seizure reduction frequency within six days of treatment initiation at 15 mg/kg/d de CBD, with a more important improvement in seizure reduction at 10 days, at a 25 mg/kg/d dose. Sixty four percent of patients reported side effects within two weeks of dose titration. More common side effects were: drowsiness, hyporexia and diarrhea, which was resolved within four weeks in about half of the patients^46^.

**Figure 2.**
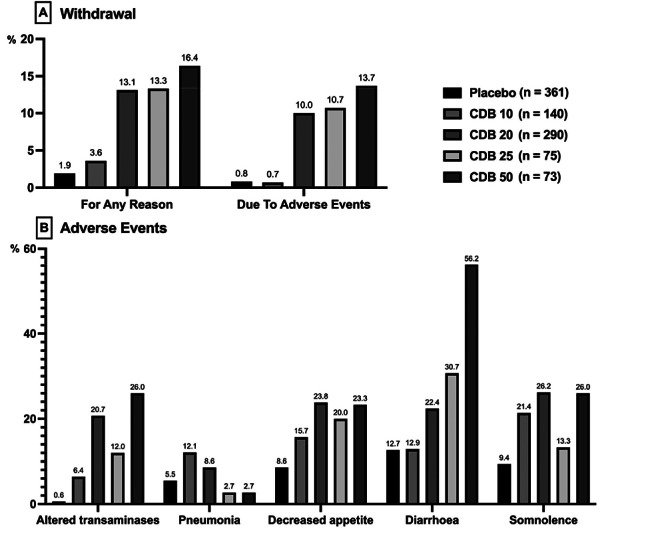
Tolerability profile of Cannabidiol (CBD) during the 14-week-treatment of the five randomized cannabidiol clinical trials in patients with Tuberous Sclerosis Complex (TSC), Lennox-Gastaut Syndrome (LGS) and Dravet Syndrome (DS). Doses: 10 mg/kg/d (CBD 10), 20 mg/kg/d (CBD 20), 25 mg/kg/d (CBD 25) and 50 mg/kg/d (CBD 50). A) Withdrawal analysis; B) Serious adverse events (altered transaminases and pneumonia) and more common adverse events (decreased appetite, diarrhea, drowsiness)

Data regarding CBD efficacy as add-on therapy in Dravet, LGS and tuberous sclerosis are presented in Figure 1. Data regarding tolerability of CBD in the five trials are presented in Figure 2. 

## EVIDENCE IN OTHER EPILEPTIC SYNDROMES

Open-label trials with less stringent methodology suggest possible benefits for epileptic encephalopathies, such as Doose and Aicardi syndromes, CDKL5 deficiency and Dup15q^47^ ^-^ ^50^. A randomized controlled trial for CBD in infantile spasms (GWPCARE7) is currently in the data analysis phase^51^. 

 In adult patients with focal refractory epilepsy, CBD studies are scarce and evidence for CBD efficacy is still lacking^52^. Fragmentary evidence is based on observational studies, case reports and subgroup analysis. CBD doses ranged from 5-50 mg/kg/day, with improved seizure control. Among different etiologies CBD use has been reported in Sturge-Weber syndrome, focal cortical dysplasia, lissencephaly, brain tumor-related epilepsy, and frontal and temporal lobe epilepsy of unknown etiology. In the emergency setting and status epilepticus treatment, benefit has been reported in children with febrile infection-related epilepsy syndrome (FIRES)^53^ and in a case report of a young man with new-onset refractory status epilepticus (NORSE)^54^ presented significant improvement that was temporally related to CBD initiation. 

 Adverse effects profile does not differ from those reported on the DS and LGS trials. Reported side effects include: drowsiness, gastrointestinal symptoms, hyporexia and weight loss^47^ ^-^ ^50^. 

## EVIDENCE FOR SYNTHETIC CANNABIDIOL

Epidiolex® is the only Cannabidiol-based medication used in randomized controlled clinical trials. Epidiolex® is a purified product directly extracted from the *Cannabis* plant. There is a current debate regarding the efficacy of synthetic CBD. An open-label study showed similar efficacy and safety profiles^55^ ^,^ ^56^. A more widespread use of synthetic CBD is still viewed with hesitancy. Proponents for natural CBD argue for a possible “entourage effect”, that proposes that different *Cannabis* components could enhance antiepileptic effect, not properly assessed in current studies. Some put forward the argument that plant-derived *Cannabis* would be more natural, with fewer side effects^57^. Synthetic Cannabis may be more appropriate for large-scale production, possibly with less environmental impact. 

## CURRENT BRAZILIAN STATUS

Federal Medicine Council (Conselho Federal de Medicina) resolution number 2113 of October 30th, 2014 set the rules for compassionate CBD use as a medical therapy, restricted to the treatment of childhood and adolescence epilepsy refractory to conventional therapies^58^. Individuals or their legal proxies were allowed to import CBD for personal use, under prescription of a licensed professional. The patient was required to fill in a form in the National Sanitary Agency (Anvisa). Due to the morosity of paperwork for importation licensing and to high demand, Anvisa authorized production and utilization of cannabinoid medications in Brazil in 2019, but did not register cannabinoids as a medication, but rather as a product under different regulatory rules^59^. Brazil followed similar models as other countries, such as Canada, Germany, Portugal and Australia with the intent of allowing faster and more widespread access to the population. Brazil did not register cannabidiol as a medication, but as a Cannabis product. A Collegiate Board Resolution 327/2019 created this novel category to allow for a faster approval of quality products, which are subject to all requirements for approval as a medication, except for the requirement of complete safety and efficacy data. These products are not subject to price control by the Anvisa Medication Market Regulation Chamber. In order to receive approval to commercialize in the Brazilian Medication Market, the pharmaceutical industry must apply for a special authorization adhering to prerequisites such as holding a Good Practice Certificate for the Production of Medications. Prati-Donaduzzi was the first pharmaceutical company to be authorized to produce and commercialize cannabidiol in Brazilian pharmacies. Cannabis products, composed exclusively of *Cannabis sativa-*derived products, must predominantly contain CBD, with no more than 0.2% tetrahydrocannabinol (THC). CBD concentrations above 0.2% should be destined to patients under palliative care, in clinically irreversible or terminal conditions. Other requirements for approval as a product include sanitary license to manufacture and import limited to five unextendable years. Imported Cannabis products must be approved for use in their country of origin and a box warning reading “this product was not evaluated for efficacy and safety by Anvisa” is required, thus placing responsibility for efficacy and safety upon the prescribing physician and the manufacturer, and requiring that the patient or legal proxy sign an informed consent form. Prescription requires a special controlled medication formulary, and can be acquired in regular pharmacies^59^.

In the Brazilian Public Healthcare System (Unified Health System - SUS), the Clinical Protocol and Therapeutic Directives - Protocolo Clínico e Diretrizes Terapêuticas (PCDT) for epilepsy, published in 2018, included the following anti seizure medications: sodium valproate, carbamazepine, clobazam, clonazepam, ethosuximide, phenytoin, phenobarbital, gabapentin, lamotrigine, levetiracetam, primidone, topiramate and vigabatrine^60^. Cannabis products are not listed in the medication list offered by SUS at no cost. The National Commission for Technology Incorporation - Comissão Nacional de Incorporação de Tecnologias (CONITEC) - following an intensive study of current evidence and cost-benefit analysis of the only available Cannabis product licensed at the time for commercialization in Brazil recommended to SUS against incorporation of this CBD product in 2022. Three studies from 2017 and 2018 on efficacy and safety - GWPCARE3 and CWPCARE4 involving Lennox-Gastaut patients and GWPCARE1b involving Dravet patients, were evaluated^61^. Cost-benefit studies were performed with an estimate cost of 1850.41 Brazilian Reais (including taxes), or approximately US$379.95 for a 30ml 200mg/ml vial at an annual cost of R$74,865 per patient (US$15,373), with an annual budget impact of 70 to 80 million reais (US$14 to 16 million). Cannabidiol received a 0.32 QALY value (quality-adjusted life year - in which 1 QALY indicates one year of perfect healthy life). Cannabidiol currently presents a higher cost than standard treatment, with an investment of 1600 Brazilian Reais (approximately US$334) to prevent an epileptic seizure and R$ 3,6 million or US$764,000) for each QALY point increase - well above standard QALY of 0,7 to 3 per capita Gross National Product PIB required for approval of a medication in the public National Health System in Brazil. Additionally, variability of Cannabis products presentation, lack of proof of equivalence and interchangeability between products and those used in clinical trials, as well as uncertainty regarding efficacy and Cannabis products magnitude of effect for the proposed use were further limitations for approval for Cannabis products in the Brazilian Public System.

In the Private Healthcare System in Brazil, CDB products are still not included in the National Agency for Supplementary Healthcare Mandatory coverage procedures for Healthcare plans.

 Internationally, cannabidiol use for refractory epilepsy was evaluated by Agencies in England (National Institute for Health and Care Excellence - NICE), Scotland (Scottish Medicines Consortium - SMC) and Argentina (Administracion Nacional de Medicamentos, Alimentos y Tecnologia Médica - ANMAT)^43^. Following approval of *Epidiolex*, these agencies strongly recommended its exclusive use for Dravet^38^ ^,^ ^39^ and Lennox-Gastaut syndromes^38^ ^,^ ^40^. 

 As of February 2022, in Brazil, 14 Cannabis-derived products can be manufactured and commercialized in pharmacies, including: Canabidiol Prati-Donaduzzi (20 mg/mL; 50 mg/mL and 200 mg/mL); Cannabidiol NuNature (17,18 mg/mL); Cannabidiol NuNature (34,36 mg/mL); Cannabidiol Farmanguinhos (200 mg/mL); Cannabidiol Verdemed (50 mg/mL); Cannabis sativa Extract Promedio (200 mg/mL); Cannabis sativa Extract Zion Medpharma (200 mg/mL); Cannabidiol Verdemed (23,75 mg/mL); Cannabis sativa Extract Alafiamed (200 mg/mL); Cannabis sativa Extract Greencare (79,14 mg/mL); Cannabis sativa Extract Ease Labs (79,14 mg/mL); Canabidiol Belcher (150 mg/mL); Cannabidiol Aura Pharma (50 mg/mL); and Cannabidiol Greencare (23,75 mg/mL)^62^. Efforts have been made to facilitate the import of a 288 Cannabidiol product list, including Epidiolex, through an online form^63^.

 The only study involving the Brazilian population was carried out in 1980. This study evaluated 15 patients with at least one focal temporal lobe seizure per week evolving to bilateral tonic clonic seizures for at least one year. Patients were randomized to receive 200-300 mg/day of cannabidiol (manufactured in Israel). Four patients showed a marked improvement in an up-to-18-week follow-up. Limitations include the fact that criteria for refractory epilepsy were not established at the time, and medications used included primidone, phenobarbital, phenytoin and clonazepam, some in monotherapy. Doses were not presented in the paper, hindering evaluation as to whether anti seizure medication dosage was optimized^12^. No other population observational or interventional study has been performed in Brazilian subjects so far. 

For literature searches, we employed descriptors chosen based upon scientific-technical MeSH (Medical Subjective Heading) and DeCS (Descriptor in Health Sciences) terms, such as “Cannabis”, “Cannabis sativa”, “Cannabidiol”, “Epilepsy” and “Brazil”, in English, Portuguese and Spanish. To our knowledge, there is currently only one ongoing clinical trial with Brazilian subjects (NCT02783092) recruiting two- to 18-year-old patients with refractory epilepsy fulfilling ILAE (International League Against Epilepsy) criteria^64^. This study will evaluate clinical response to purified CBD available in Brazil, at 5-25 mg/kg/day doses. This study was initiated in 2019, with completion foreseen in the fourth quarter of 2021^64^.

## PROPOSED ALGORITHM FOR CBD USE IN REFRACTORY EPILEPSY

**Figure 3.**
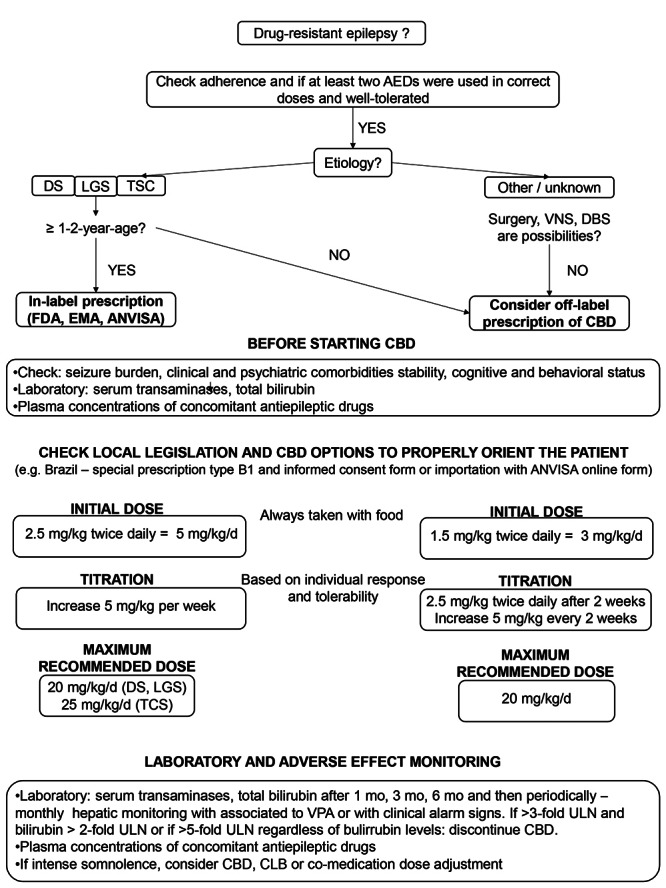
Algorithm for use of CBD in epilepsy.

Adapted from von Wred R et al (2021)^65^.AEDs: antiepileptic drugs; DS: Dravet Syndrome; LGS: Lennox-Gastaut Syndrome; TSC: Tuberous Sclerosis Complex; VNS: vagus nerve stimulation; DBS: deep brain stimulation; FDA US: Food and Drug Administration; EMA: European Medicine Agency; ANVISA: Brazilian Health Regulatory Agency; CBD: cannabidiol; VPA: valproate; CLB: clobazam**;** ULN: upper limit of normal.

A proposed algorithm for CBD use in medically refractory epilepsy is presented in Figure 3 ^65^. 

In conclusion, epilepsy burden, especially medically refractory epilepsy, affects patients and families, particularly for medically refractory epilepsy^66^. CBD offers hope in treating epilepsy. Current evidence demonstrates efficacy and safety in prescribing CBD for Dravet and Lennox-Gastaut syndrome and Tuberous Sclerosis Complex. Newer randomized studies are currently underway to evaluate CBD use in other epilepsy types. Caution is advised against indiscriminate CBD use. CBD has a powerful marketing appeal that may risk foregoing significant lacunae in scientific knowledge that need to be clarified and better understood in order to delineate rational CBD use in epilepsy. Medically refractory epilepsy encompasses a heterogenous group of conditions, and currently available RCT results should not be automatically extrapolated to other epilepsy conditions. 

 CBD access is hindered by elevated costs that cannot be afforded by the majority of the Brazilian population. In addition to acquisition of further scientific knowledge regarding Cannabis products, public healthcare policies must be implemented to allow widespread access to Cannabis products in Brazil and worldwide. Production of purely synthetic products with equivalent efficacy compared to natural *Cannabis* products may decrease production cost, and, consequently, price. Recently approved legislation and resolutions should facilitate a growing scientific knowledge of Cannabis-derived products, as well as facilitating promotion of strategies to a more widespread use of these products. 

Lastly, current CBD prescription patterns raise concerns as to indiscriminate use. Individual clinical and pharmacological responses must be strictly evaluated to allow scientifically-based CBD use. Careful dose titration and side-effects monitoring are key to maximizing tolerability of Cannabis products. Patient and family expectations of treatment results (sometimes previously artificially inflated) must be adjusted in order to promote better quality of life for people with medically refractory epilepsy. 
